# Structure of Shock Wave in Nanoscale Porous Nickel at Pressures up to 7 GPa

**DOI:** 10.3390/ma15238501

**Published:** 2022-11-29

**Authors:** Alexander Dolgoborodov, Timofei Rostilov, Sergey Ananev, Vadim Ziborov, Leonid Grishin, Mikhail Kuskov, Alexey Zhigach

**Affiliations:** 1 Joint Institute for High Temperatures, Russian Academy of Sciences, Izhorskaya 13 Bd. 2, 125412 Moscow, Russia; 2 N. Semenov Federal Research Center for Chemical Physics, Russian Academy of Sciences, Kosygin St. 4, 119991 Moscow, Russia

**Keywords:** shock wave, compaction wave, porous, nanoparticle, nickel, high velocity impact, precursor wave, Hugoniot, VISAR

## Abstract

The structure of shock waves in pressed porous samples of nickel nanoparticles was investigated in a series of uniaxial planar plate impact experiments in the pressure range of 1.6–7.1 GPa. The initial porosity of the samples was about 50%. Wave profiles were obtained using laser velocimetry techniques. The nanomaterial demonstrated a complex response to shock loading including the development of a two-wave structure associated with precursor and compaction waves. The effect on profiles and measurements of the observed precursor reverberations propagating between the front of a compaction wave and a monitored sample surface was described. The obtained wave profiles were used to estimate the thicknesses of precursor and compaction wave fronts.

## 1. Introduction

The complexity of the shock compression (or the shock compaction) process of porous media is associated with rapid pore collapse occurring in a shock wave front and the broadening of a wave front [1,2]. At relatively low and moderate pressures, the pore collapse process strongly affects shock compression. A wave propagating at these conditions is called a compaction wave due to the leading role of this process. A compaction wave may be accompanied by an outrunning precursor wave, causing a slight compression of a material [3].

An understanding of the shock compression of granular materials is important due to their use in many scientific and engineering applications: shock wave pressing and synthesis, blast wave and hypervelocity impact mitigation, and so on [4,5,6]. The process of the shock compression of porous materials is much more complicated and much less clear compared to solid materials, since processes arising during compaction are related not only to the internal material properties, but also to the sample morphology (particle size, shape, strength, etc.).

The effect of particle size on the shock wave structure has been well-studied [2,7,8,9]. A comparative analysis of shock compression data for nano-and microporous media may reveal the effect of particle size on the shock compression process more effectively than an analysis of two different microporous materials. It also can highlight unique features of both types of porous media.

In our previous work, we demonstrated that the Hugoniot of pressed nickel nanoparticles with a porosity of 50% and an average particle size of 50 nm contains no features attributable to the nanostructure and coincides with the Hugoniot of microporous Ni of the same porosity at pressures up to 61 GPa [10]. Moreover, the development of a complex two-wave structure in nanoporous Ni samples at low pressures was also observed.

The purpose of the present study was to investigate this feature and describe the precursor and compaction wave fronts in terms of the strain rate, wave front rise time, and thickness. These experimentally defined parameters of the shock compaction of nanoporous metal could provide a basis for computational works because wave fronts are considered in simulations [5,6,11,12]. Generally, experimental data are needed to develop novel theoretical models to describe the behavior of materials under dynamic loading [13].

Nickel nanoparticles also serve as constituents in reactive powder mixtures. Previous works have revealed the shock properties of micro- and nanoscale Ni + Al powders and characteristics of the shock-induced NiAl intermetallic formation [5,14,15,16,17]. The complete analysis of the shock compressibility of such powder mixtures requires knowledge of the shock wave propagation parameters of its components including data for the nanosized nickel powder.

## 2. Material Properties and Samples

**Figure 1:**
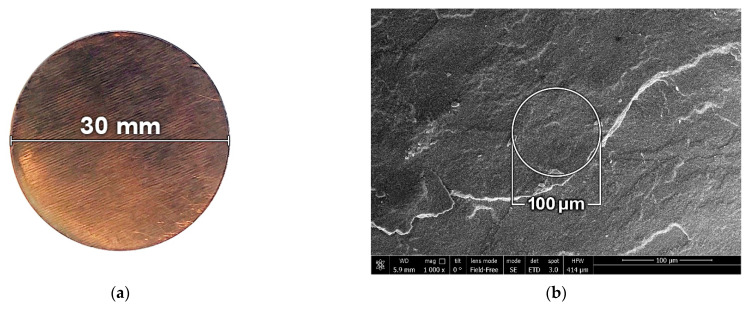

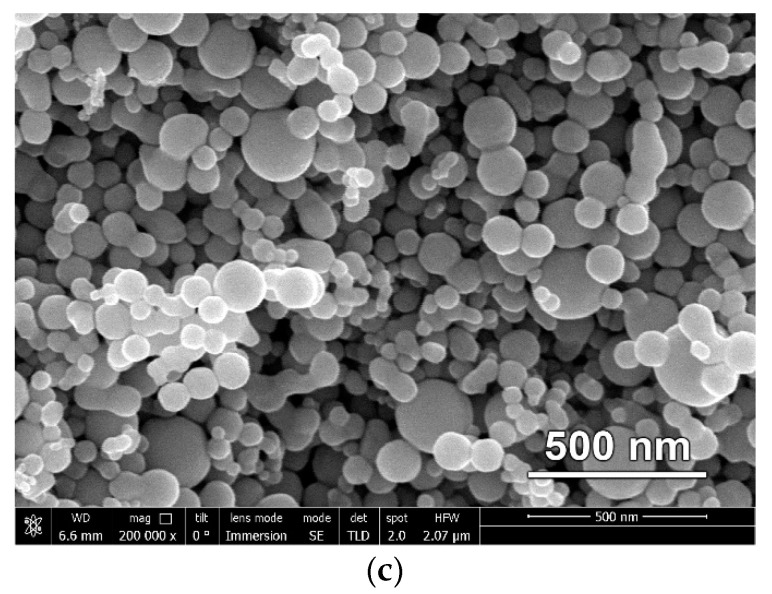
(**a**) Sample of the pressed nNi. (**b**) Microstructure of the pressed nNi. The circle illustrates the approximate size of the VISAR monitoring point. (**c**) Nanostructure of the pressed nNi.

The material studied has been thoroughly described in our previous work [10]. Briefly, nanosized nickel particles (nNi) were synthesized using the flow-levitation method [18]. X-ray diffraction analysis demonstrated that the particles consisted primarily of metallic nickel with a small NiO_x_ oxide content of less than 0.3% weight. The nickel nanopowder was pressed in an argon-filled chamber using a hydraulic press to prevent the negative effect of nickel oxidation, which may occur in the case of cold pressing [10]. A uniaxial pressure up to 2700 MPa was applied to compact the powder into samples in the form of tablets (disks) of 30- or 40-mm in diameter (Figure 1a). The initial sample porosity was controlled by continuous monitoring of the applied pressure and the sample thickness. After pressing, the tablets were removed from molds and placed in an air atmosphere. The following process of nickel oxidation on the particle surfaces heated the samples by 50 °C. The weight of the tablets increased by about 2% within 3 h and then remained unchanged. A photo of the sample and scanning electron microscope images of the micro- and nanostructure of the material are presented in Figure 1. The spherical particles had an average size of approximately 50 nm (Figure 1c).

The final sample thicknesses *h_s_* are listed in Table 1. The initial sample density *ρ*_0_ varied from 4.5 to 4.7 g/cm^3^ due to the inability to create absolutely identical samples; the average density value was 4.62 g/cm^3^. The initial sample porosity ε ranged from 47 to 49%, this value was calculated as $\varphi =\left(1-\rho_{0}/\rho_{s}\right)\cdot 100\%$, where *ρ_s_* is the solid density of nickel.

## 3. Experimental Setup

**Figure 2:**
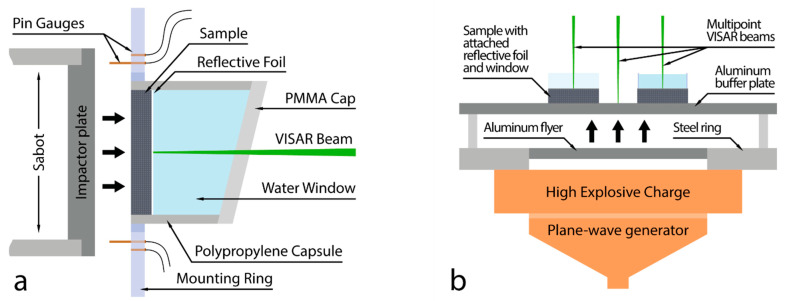
(**a**) Schematic of the powder gun experiment. (**b**) Schematic of the explosive charge experiment.

The experimental facilities used in the present work were the same as those described in [10,19]. Experiments were carried out using a powder gun or chamber for explosive charges, both located at the JIHT RAS. A powder gun with a 57-mm diameter bore provided uniaxial planar plate impact experiments with impactor velocities V_i_ below 1 km/s (Experiments 1–5 in the Table 1, Table 2 and Table 3). This experimental setup is shown schematically in Figure 2a. The cylindrical experimental assemblies consisted of a sample, a 7-μm-thick aluminum reflective foil, and a window.

The VISAR interferometer technique [20] with a time resolution of ~1 ns was employed in all experiments to record the velocity profiles of the sample–window interface (more precisely, of the foil–window interface) at the moment of the shock wave arrival.

The system of coaxial shorting pin gauges provided measurements of the impactor velocity V_i_ and impact tilt (i.e., the angle between the impactor frontal surface and the sample surface facing it). The precision of V_i_ measurements was about 1%. The impact tilt was typically to the order of 2 mrad for the gun experiments.

The mounting ring with the installed assembly and pins was placed into the experimental chamber of the gun facility. Before each shot, the chamber was evacuated to prevent a negative air influence on the measurements.

Explosive generators were employed to conduct shots with impactor velocities of 1 km/s and above (Figure 2b). An aluminum impactor (flyer) was accelerated by a detonation of high explosive charges. The required velocity of the impactor was set by varying its thickness (7–10 mm). The impactor collided with the 2 mm aluminum buffer plate and generated a shock wave in the samples glued on the opposite side of the plate. The 10–100 μm thick aluminum foils were glued on the rear sample surfaces to ensure the reflection of the probing laser beam. Reflective foils prevent the loss of laser reflection during the formation of “jets” of material upon the movement of the monitored surface. The thickness of the foil was selected depending on the speed of the impactor. Up to three samples with different windows (water or LiF) were installed in each experiment. After reflection, the laser beams entered the four-channel VISAR. One of the probing beams was reflected from the surface of the buffer plate to provide a measurement of the shock wave arrival time at the buffer–sample interface. The buffer plate also simplifies conducting multi-sample experiments.

Windows reduce the negative effect of porous sample destruction under shock wave loading on the VISAR measurements [21] and define the picture of shock wave reverberations occurring in a sample. Due to the shock-induced changes in refraction index, the use of the LiF window requires corrections for VISAR measurements that were made as described in [22].

The window material, reflective foil thickness, the impactor material, and velocity for each shot are listed in Table 1.

Figure 1b illustrates the monitoring point of VISAR (approximately 100 μm in diameter) covering the surface of the nNi sample. This point is much larger than the characteristic particle size of 50 nm. Thus, a unique composition of particles and voids in the vicinity of the monitoring point and in a material below this point should have less effect on the results of a particular experiment, than in the case of a material with microsized heterogeneities [2,19].

## 4. Results

**Figure 3:**
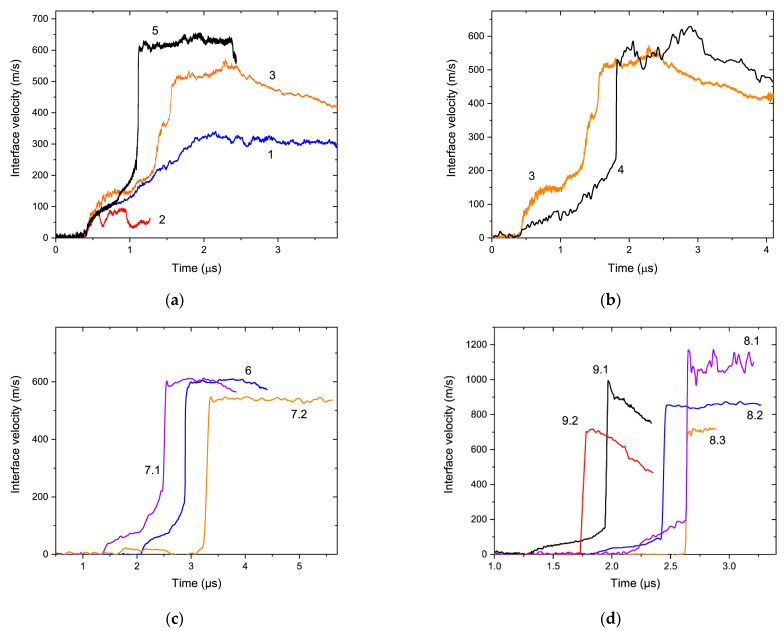
Measured velocity profiles. The numbers correspond to the experiment numeration (see Table 1, Table 2 and Table 3). (**a**) Experiments 1–3, 5 were conducted with the nearly same sample thickness, but different impact velocities. The water windows were used in Experiments 1, 3, 5; the LiF window was used in Experiment 2 (only the precursor trace was available). Time t = 0 was arbitrary, the profiles were adjusted to coincide in the precursor fronts. (**b**) Experiments 3 and 4 were performed with different sample thicknesses and the nearly same impact velocity. Time t = 0 was arbitrary, the profiles were adjusted to coincide in precursor fronts. (**c**) Experiments 6, 7.1, and 7.2 were conducted with the same impact velocity, but different sample thickness as the windows were water (Experiments 6, 7.1) and LiF (Experiment 7.2). Time t = 0 was the time of impact. (**d**) The sample thickness and impact velocity were the same as in the Experiment 8 set, but the windows were different; the same applied to set 9. Time t = 0 was the time of impact.

The measured wave profiles are presented in Figure 3. The forward portions of most profiles had notable multistep shapes, especially in Experiments 1, 3, 5, 6, and 7.1. These shapes indicate the development of a significant two-wave structure associated with propagation of the precursor and compaction waves, and with reverberations of the precursor between the sample–window interface and the front of the oncoming compaction wave. For example, in Experiment 5 (Figure 3a) the first rapid velocity jump to ~70 m/s at ~0.45 μs was associated with the precursor arrival, the next one, the velocity jump to ~140 m/s at ~0.85 μs, was associated with the precursor reverberation, and the biggest velocity jump to ~610 m/s at ~1.1 was associated with the compaction wave arrival. These jumps were separated by the gradual velocity increases.

Parameters of the precursor and compaction wave fronts listed in Table 2 and Table 3. Subscripts 1 and 2 in the values of particle velocity *u_p_*, pressure *p* and wave velocity *U_s_* refer to the precursor and compaction waves, respectively. The Hugoniot data from Experiments 6 and 7 were previously presented in [10]; here, we provide the data for the precursor and compaction waves for each sample in these shots, while it was summarized in the form of average values in [10].

The arrival of the compaction wave onto the sample–window interface produced the largest velocity jump on a profile followed by a plateau containing some velocity fluctuations. A subsequent rapid decrease in velocity was caused by a release wave originated at the rear surface of impactor, for example, at ~2.4 μs in Experiments 3 and 5 in Figure 3. In some cases, the release waves partly overtook the compaction waves (Experiments 9.1 and 9.2 in Figure 3d).

Wave velocities were calculated using the time of impact and half-amplitude points of the wave fronts obtained by the analysis of the VISAR profiles. In experiments with low impactor velocities, reverberations complicate the calculations of the compaction wave velocities, especially in Experiment 1. Due to the distortion of the compaction wave fronts by the intense reverberations, it was not clear where exactly the half-amplitude points were. This feature negatively affected the accuracy of the measurements. Uncertainties in the precursor and compaction wave velocities were estimated to be to the order of 5%.

The Hugoniot data were obtained using the impedance matching method considering the two-wave structure [23,24,25]. Certain aspects of our approach will be highlighted in the discussion section.

**Figure 4:**
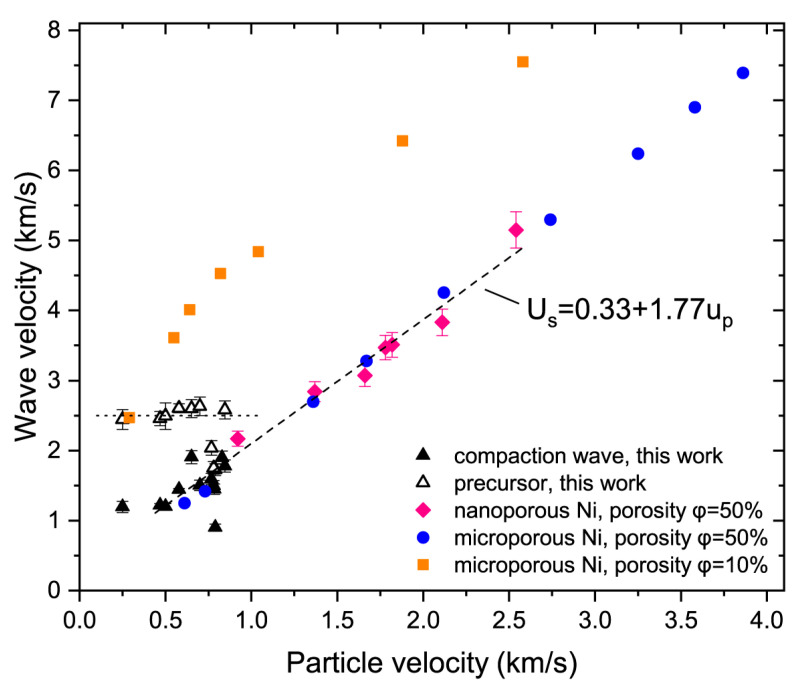
Hugoniot of nanoscale porous nickel on the shock wave velocity–particle velocity plane. Earlier points for nanoporous Ni [10] and microporous Ni [26] are also plotted.

The Hugoniot data for pressed nNi were in close agreement with that of the microporous Ni of the same porosity (Figure 4). The dependence between overall compaction wave velocity $U_{s}$ and particle velocity $u_{p}$ for the pressed nNi may be approximated by the linear relation (0.5 < *u_p_* < 2.5 km/s):

(1) $$U_{s}=0.33+1.77u_{p}\;\left[\mathrm{km}/s\right],$$

The point with the lowest particle velocity (Experiment 4) is inconsistent with other data. This may indicate a decrease in the Hugoniot slope in the low particle velocity region or show the effect of sample-to-sample variations causing scatter in the data. The Hugoniot data for microporous nickel (porosity *ε* = 10%) demonstrate an increase in the slope in this region [26] (Figure 4), while the opposite situation may also be observed in the porous media [27]. The point with the lowest compaction wave velocity (Experiment 7.2) probably demonstrates that properties of some samples distinctly differed from others.

The average precursor velocity was found to be 2.5 km/s, but several data points deviated significantly from this value. In Figure 4, the wave velocities *U_s_*_1_ were drawn versus the particle velocities *u_p_*_2_. Such representation demonstrates that precursors should not be registered at *u_p_* > 1.25 km/s because the compaction waves must be faster than the precursors at these conditions.

While the shocked state of a material may be described by its Hugoniot, the transition to this state and the wave front structure may be characterized by a maximum longitudinal strain rate $\dot{\varepsilon }$ (strain rate further in the paper) occurring in the wave front and by the wave rise time τ.

**Figure 5:**
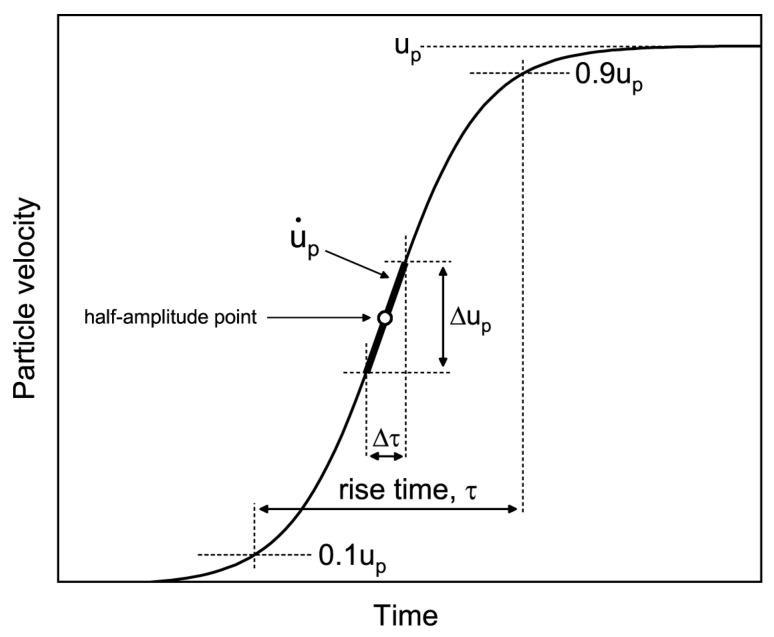
The idealized particle velocity profile demonstrating the calculations of the maximum particle velocity gradient and rise time. The thick line segment had a slope equal to a maximum particle velocity gradient. $u_{p}$ is the particle velocity behind a wave front.

**Figure 6:**
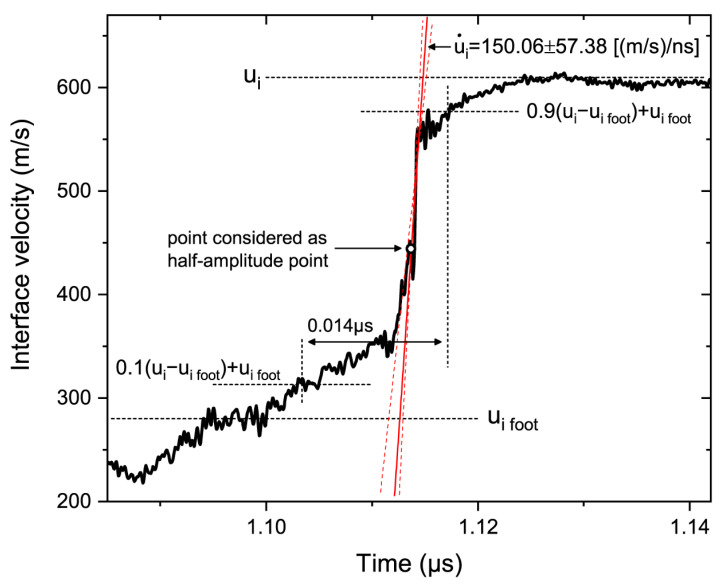
Actual calculations of the wavefront parameters in Experiment 5. The red solid line had a slope equal to the maximum velocity gradient; the dashed lines demonstrate the upper and lower boundaries of this value. The foot of the compaction wave chosen was somewhat arguable. The measured impact tilt in the experiment was 0.7 mrad.

The rise time *τ* was determined by utilizing the 10–90% section of the total velocity profile amplitude *u_p_* [2,28,29] (see Figure 5 and Figure 6), though some authors used the 5–95% section [30,31] or another [32]. The value of rise time can vary greatly depending on the approach [33,34].

The strain rate was calculated using the formula [35]:

(2) $$\dot{\varepsilon }={\dot{u}}_{p}/U_{s},\;$$

where $U_{s}$ is the wave velocity and ${\dot{u}}_{p}$ is the maximum particle velocity gradient. The latter value can be found as ${\dot{u}}_{p}=\Delta u_{p}/\Delta \tau$, where $\Delta u_{p}$ is the velocity rise in the fastest rising portion of the wave front and Δτ is the portion duration (Figure 5).

In our calculations, the compaction wave velocity *U_s_*_2_ was obtained as

(3) $$U_{s2}=\left[h_{s}+{\bar{u}}_{i}\left(t_{2}-t_{1}\right)\right]/\left(t_{2}-t_{0}\right)\;$$

where ${\bar{u}}_{i}$ is the average velocity of the sample-window or free surface after the precursor arrival and before the compaction wave arrival, $t_{0}$ is the impact time, $t_{1}$ is the time of precursor arrival, $t_{2}$ is the time of compaction wave arrival. Equation (3) only implies that a compaction wave overtakes the interface accelerated by a precursor to the average velocity ${\bar{u}}_{i}$. Thereby, the obtained values of $U_{s2}$ “include” particle velocities behind a precursor wave front *u_p_*_1_. This fact should be considered in the ${\dot{\varepsilon }}_{2}$ calculations

(4) $${\dot{\varepsilon }}_{2}={\dot{u}}_{p2}/\left(U_{s2}-u_{p1}\right)\;\;$$

We used Equations (3) and (4) to obtain the values of ${\dot{\varepsilon }}_{1}$ and ${\dot{\varepsilon }}_{2}$, respectively.

The measured profiles (Figure 3) were not equal to the particle velocity profiles due to the shock impedance mismatch between the sample and window materials or another reason in the case of the free surface [36]. These only allow for the evaluation of the maximum interface velocity gradient ${\dot{u}}_{i}$ (Figure 6), which can be recalculated into ${\dot{u}}_{p}$ using the Hugoniot data and profiles [37].

**Figure 7:**
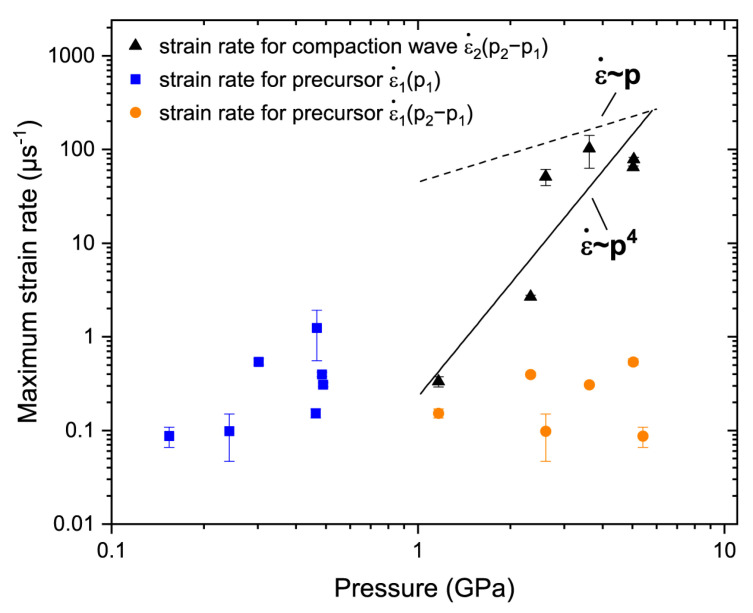
Strain rate versus pressure for the precursor and compaction waves. The strain rates for the precursor ${\dot{\varepsilon }}_{1}$ and compaction wave ${\dot{\varepsilon }}_{2}$ were obtained by Equations (2) and (4), respectively, only for experiments with 7- and 10-μm thick reflective foils. *p*_2_ − *p*_1_ is the pressure rise only in the compaction wave, and *p*_1_ and *p*_2_ are the pressures behind the precursor and compaction wave fronts, respectively. The points ${\dot{\varepsilon }}_{1}\left(p_{2}-p_{1}\right)$ are drawn only for experiments where the parameters for the compaction wave are available; such representation illustrates how ${\dot{\varepsilon }}_{1}$ depends on the applied pressure.

The measurements of the wave front parameters were complicated by the precursor reverberations and other experimental or material related features (for example, the possible partial breakage of the reflective foil by particles in some cases). The frontal portions of profiles contained some velocity oscillations and did not have ideal sigmoidal shapes. It was not always clear which profile portion exactly represents ${\dot{u}}_{i}$, especially in the cases when reverberations strongly interfered with compaction wave fronts (Experiments 1 and 3). We estimated the upper and the lower boundaries for the values of ${\dot{u}}_{i}$, that could differ by several tens of percent, and determined an average between them (Figure 6). The error bars shown in Figure 7 represent the uncertainty of such estimations.

The strain rates and rise times were obtained only for experiments with the 7- and 10-μm thick reflective foils because even relatively thin reflective layers may negatively affect the profile records [38].

The correct analysis of wave front parameters such as rise times and strain rates is possible if shock waves reach steady states [35,39]. Propagation distances at which waves become steady for each studied regime was not determined in this work, thus, the wave front data presented here had an approximate character.

The strain rate arising in the front of a steady shock wave scales with the peak pressure as

(5) $$\dot{\varepsilon }\sim p^{n}\;\;$$

where the exponent n typically lies between 1 and 4 and depends on the material type [35,39,40].

In the case of the two-wave structure, the pressure rise only in the compaction wave *p*_2_ − *p*_1_ should be used to build a relation in the form of Equation (5) [35]. The exponent n for nNi is roughly equal to 4 (Figure 7). It is interesting that this value is typical for solid metals and not for powders. In Figure 7, the values of ${\dot{\varepsilon }}_{1}$ were also plotted versus *p*_2_ − *p*_1_; this representation shows that the values of ${\dot{\varepsilon }}_{1}$ do not noticeably scale with the pressure increase.

We suppose that the rise time calculations require a compaction wave front to be undisturbed by reverberations. Thereby, the values of *τ*_2_ were not presented for Experiments 1 and 3. Additionally, due to the reverberations and the velocity rise behind the precursor wave front, it was sometimes hard to define the velocity level related to the foot of the compaction wave (see $u_{i\;foot}\;$ in Figure 6). Thus, in some cases, the measurements of compaction wave rise times were based on somewhat arbitrary choices of $u_{i\;foot}$.

**Figure 8:**
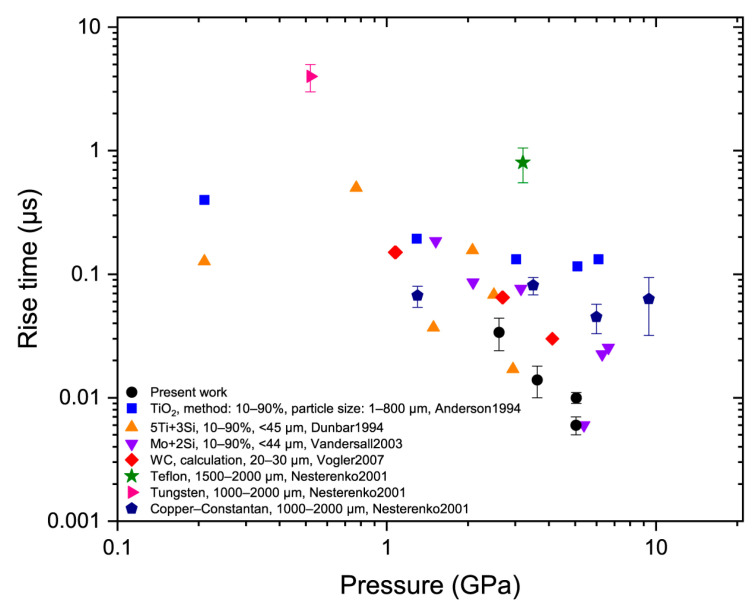
Compaction wave rise time versus pressure. The rise times for nNi are plotted versus the pressure rise only in the compaction wave *p*_2_ − *p*_1_. Earlier data from [28,29,33,34,41] are also plotted.

Figure 8 shows that the measured compaction wave rise times were comparable to those obtained for the micropowders. The experimental points for nNi were located in the lower part of the area characteristic of micropowders, while some points for powders with 1–2 mm particles lay noticeably above this area.

## 5. Discussion

### 5.1. Precursor Reverberations

Precursor reverberations are not a unique feature of the nanomaterial tested. The same effect was observed in plate impact experiments with different materials [25,42,43,44,45]. When a precursor arrives at a sample–window interface, it may reflect back into a sample as an unloading wave or compression wave depending on the difference between the shock impedances of a sample material in the elastic range and a window material [42].

**Figure 9:**
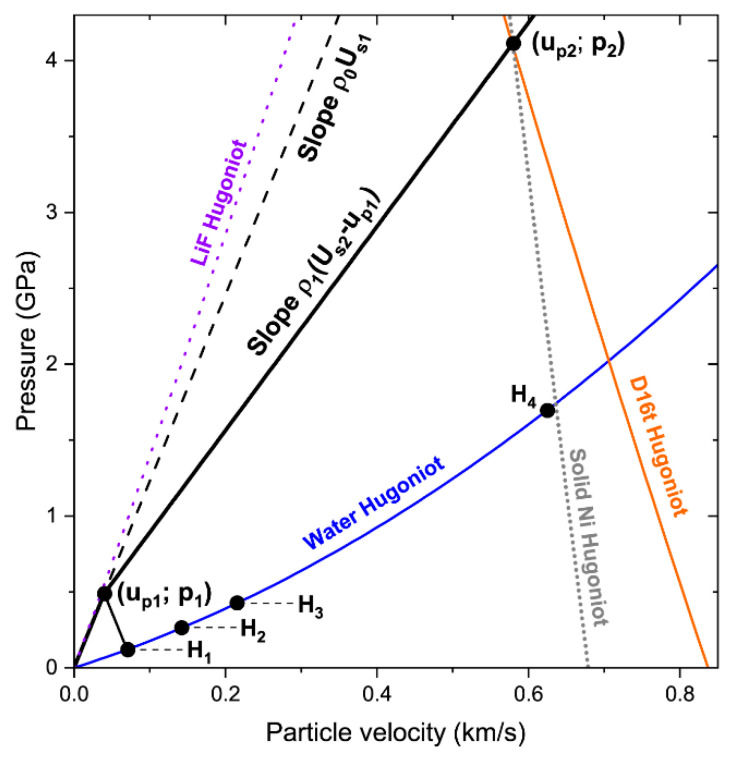
Pressure–particle velocity diagram for Experiment 5. The points H_1_–H_4_ are the shocked states in the water window after the precursor (H_1_), its reflections (H_2_, H_3_), and compaction wave (H_4_) arrivals. These were obtained using the water Hugoniot and the velocity profile (see Figure 10). The LiF Hugoniot is shown for comparison. The reflected solid Ni Hugoniot drawn from the point (*u_p_*_2_, *p*_2_) nearly crossed the point H_4_, which demonstrates that probably all pores of the sample were collapsed by the compaction wave.

**Figure 10:**
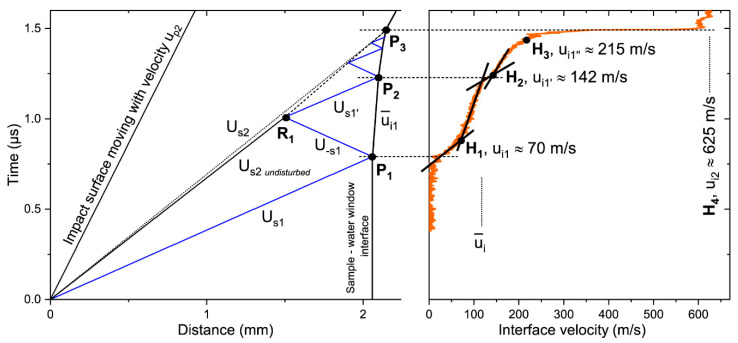
Distance–time diagram (**left side**) combined with the sample–water window interface velocity profile (**right side**) for Experiment 5. The time axis was the same for both plots, time t = 0 was the time of impact. The segment line connecting the points (0; 0) and P_1_ shows the trajectory of the precursor inside the sample, which reached the interface at point P_1_. P_1_R_1_ is the path of the first precursor reflection moving inside the sample; this reflection collides with the oncoming compaction wave in the point R_1_. R_1_P_2_ is the trajectory of the first precursor reflection moving to the interface, this reflection arrives onto it at point P_2_, producing the next reverberation cycle (not marked with points). P_1_P_2_ is the path of the interface after the initial precursor arrival and before the first reflection arrival. The compaction wave reaches the interface at point P_3_. The velocities drawn near the segment lines on the left side are the velocities of the related waves or surfaces. Wave paths in the sample behind the compaction wave, the impactor, and water window are not shown. Note that ${\bar{u}}_{i}$, ${\bar{u}}_{i1}$, $u_{i1}$ are different parameters as described in the text.

The forward parts of profiles, which are strongly affected by precursor reverberations, may be described using the simultaneous analysis of a wave profile, distance–time (x–t) and pressure–particle velocity (*p*–*u_p_*) diagrams (Figure 9 and Figure 10). Here, we provide this analysis for Experiment 5. The elastic portion of the nNi Hugoniot lies above the water Hugoniot (Figure 9). Therefore, the precursor reflected back into the sample as an unloading wave in Experiment 5 and other experiments with water windows.

The initial precursor arrival (point P_1_ in Figure 10) caused the interface acceleration to the average velocity ${\bar{u}}_{i1}$. As seen in Figure 10, right, the first rapid velocity jump was followed by the gradual velocity rise (after point H_1_) as well as the next jump (after point H_2_). This rise may be associated with work-hardening [21] or the nonuniform response of particles of different sizes to compression in a precursor. In the Hugoniot elastic limit (HEL) ($p_{1}$) calculations, we used the velocity $u_{i1}$ defined as an intersection of straight lines characterizing the rapid and gradual rises (point H_1_ in Figure 10) [46,47]. Then, the point (*u_p_*_1_, $p_{1}$) was obtained using the reflected $\rho_{0}U_{s1}u_{p}$ line.

The first interaction of the reflected precursor and the oncoming compaction wave in the point R_1_ (Figure 10) slowed down the latter wave and produced the new elastic compression wave rushing to the reflecting surface [42]. This new wave reached the surface (point P_2_ in Figure 10) and generated the next reverberation cycle. The x–t diagram (Figure 10) showed four arrivals, but even the third jump (point H_3_) was almost masked by the foot of the compaction wave. It is worth noting that $2u_{i1}\approx u_{i1^{\prime }}$, $3u_{i1}\approx u_{i1^{\prime \prime }}$. It is currently not clear how large the difference is between the states of nNi behind the initial precursor and its reverberations moving to the interface. For simplicity, here, we assumed that the compaction wave travelled all of its propagation distance in the media compressed to the state (*u_p_*_1_, $p_{1}$).

The obtained values of *U*_*s*2_ represent the average propagation velocity of the compaction wave. However, it is possible to estimate the compaction wave velocity before the first collision with the reflected precursor. We assumed that the first precursor reflection propagates into the sample with the velocity $U_{-s1}=U_{s1}-u_{p1}$ and the second elastic compression wave has the velocity $U_{s1^{\prime }}=U_{s1}+{\bar{u}}_{i1}$ [48]. Using the x–t diagram and knowing the arrival times of the latter wave and the initial precursor (points P_1_ and P_2_ in Figure 10), one may draw the paths of reflections and estimate the time and distance of the first wave interaction (point R_1_ in Figure 10). The propagation velocity of the undisturbed compaction wave evaluated in this way exceeded *U_s_*_2_ by 3% in Experiment 5. The first collision took place at a distance of ~1.5 mm from the impact surface, implying that the residual porosity of the shocked material may differ along the sample due to stepwise deceleration of the compaction wave [42].

It should be noted that the x–t diagram employing velocities $U_{-s1}$ and $U_{s1^{\prime }}$ as the second and the third reverberation cycles did not completely match the corresponding profile. Thereby, the assumptions of precursor reflection velocities may not be accurate.

**Figure 11:**
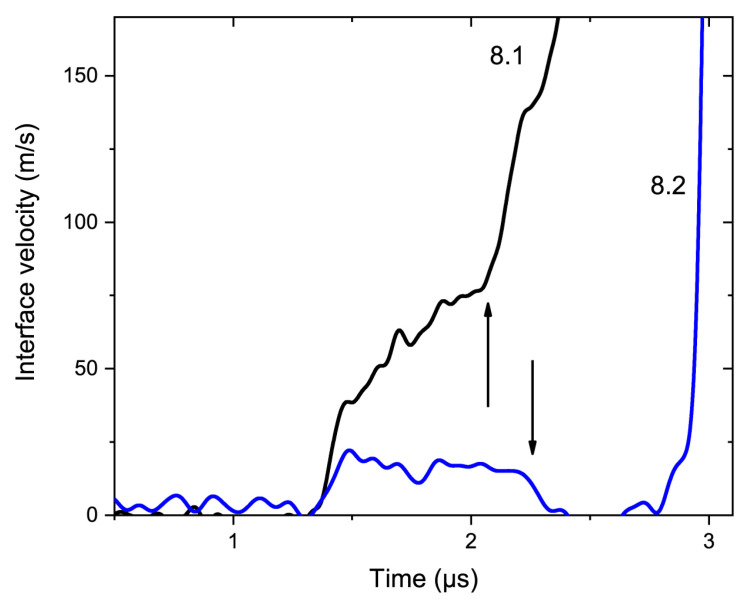
Precursors in Experiment 8. Here, time t = 0 was arbitrary, profiles were adjusted to coincide in precursor fronts. The arrows highlight the arrivals of the precursor reflections, in the case of Experiment 8.1, it was a compression wave; in the case of Experiment 8.2, it was a release wave.

The elastic part of the nNi Hugoniot may almost coincide with the LiF Hugoniot (Figure 9), but due to the scatter in precursor velocities and sample densities in some cases, reflections may occur. In experiments with LiF windows, we observed the “compression–compression–release” reflection scenario instead of the “compression–release–compression” reflection scenario. For example, in Experiment 8.2 we observed a deceleration of the monitored interface, instead of acceleration (see arrows in Figure 11).

Line H_4_ in Figure 10 corresponds to the state on the water Hugoniot (point H_4_ in Figure 9). This line marks the average value of the velocity on the plateau behind the compaction wave front. The mirrored solid nickel Hugoniot [26] can be used to satisfactorily connect the states (*u_p_*_2_, *p*_2_) and H_4_. Further description of this feature was beyond the scope of this work.

**Figure 12:**
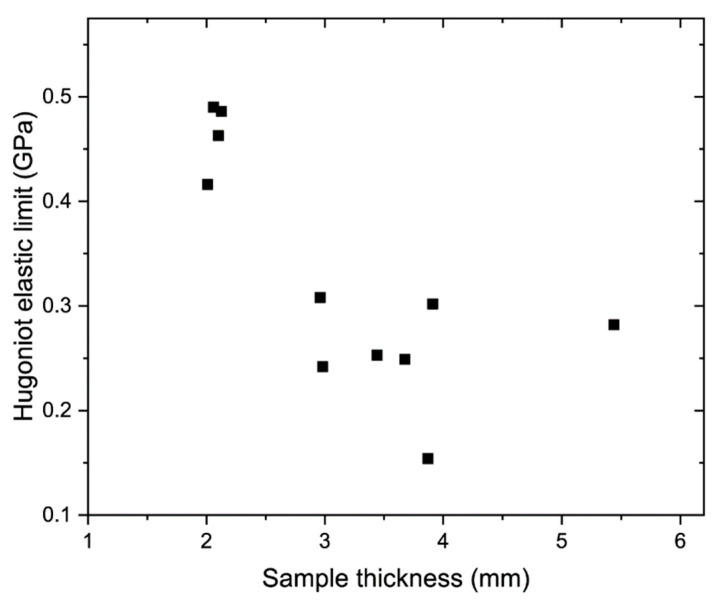
Overall Hugoniot elastic limit versus the sample thickness data points.

The overall sample thickness–HEL data (Figure 12) displayed precursor attenuation (i.e., the values of HEL were lower in experiments with thicker samples). Thus, the effect of reverberations was less significant for thick samples, for example, the compaction wave front in Experiment 4 did not contain a notable cusp compared to Experiment 3 (Figure 3b). The effect was also weaker for the stronger loading regimes due to the higher compaction wave velocities and amplitudes.

### 5.2. Aspects of Profiles and Wave Front Data

The profiles and Hugoniot data contained some features that can be attributed to the effect of sample-to-sample variations: (i) the differences in the precursor traces in the experiments with almost the same sample thickness and the same window material (e.g., in Experiment 3, the trace of precursor lies above than ones recorded in Experiments 1 and 5); (ii) the notable scatter in the precursor propagation velocities in some cases; and (iii) the overall scatter of the Hugoniot and strain rate data.

The velocity fluctuations on the plateaus behind the compaction wave fronts in Experiments 3 and 4 were probably related to the complex effects of multiple wave reverberations occurring in a shocked heterogeneous media.

Apparently, in Experiments 8.3 and 9.2, the precursors did not noticeably accelerate the investigated surfaces due to a combination of reasons: their lower than usual amplitudes were obscured by the stiff LiF windows, which made them completely indistinguishable due to the signal noise (i.e., they would probably be observed if the material of the windows was water). Another explanation of their absence is that the compaction waves were faster than the precursors in these experiments, which may also be attributed to the sample-to-sample variations.

The wave front thickness can be obtained as the product of the wave propagation velocity by the rise time. This product, divided by the average particle size, gives the number of average particles that can occupy the wave front [30]. In the calculations, we neglected that a precursor could slightly compress the particles and reduce their average size, so we used the value of 50 nm for both the precursor and compaction wave thicknesses.

**Figure 13:**
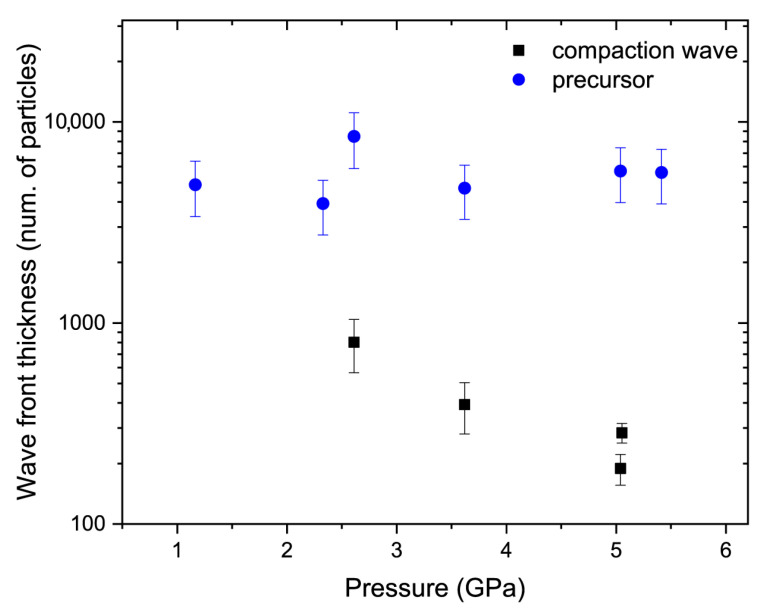
Wave front thickness versus the pressure data for both waves.

The compaction wave thickness decreased from approximately 800 average particles at 2.6 GPa to 240 average particles at 5.0 GPa (Figure 13). The pressures here and in Figure 13 show that the pressure rise was only in the compaction wave *p*_2_ − *p*_1_. The precursor wave thickness apparently does not depend on the applied pressure and its values were roughly 10 times higher than the corresponding compaction wave thicknesses.

Agreement between the rise time data for the nNi and micropowders (Figure 8) produced a great discrepancy between the compaction wave thicknesses expressed as the number of average particles. Thus, the wave thickness data obtained in this work turned out to be in contradiction with the results of the microporous studies, indicating that the wave front thickness should be comparable with the average particle size [2,29,30].

Studies [8,9] have revealed that precursors are less distinguishable in non-metallic powders with fine particles. The nanoparticles in the present study were extremely fine compared to those studied in [8,9], but the precursors were clearly visible, though the material of constituents may play a key role here. In powders, the precursor wave travels across interparticle connections and forces particles to slightly change their places [2,3], thus the material density behind the precursor front is higher than the initial density, but pores remain open until they are completely or partially closed by the compaction wave. It is worth noting that in nNi, the precursor wave front simultaneously covers thousands of particles, as shown in Figure 13. The number of particles should apparently be lower for a micropowder. Further studies of metallic and non-metallic nanoscale powders will expand an understanding of the precursor formation in such media.

As it can be seen from Figure 4, the shock stress–strain rate data for nNi may be adequately approximated by the fourth-power relation, which disagrees with the powder media strain rate data. For solid metals, the exponent n in Equation (5) was close to 4 [35], for powders n ≈ 1 [9,39,40,41]. We did not see considerable differences in the Hugoniot data for micro- and nanoporous Ni, and the rise times for the nNi and micropowders appears to be comparable. Nonetheless, the notable discrepancy in the $\dot{\varepsilon }-p$ data for the nNi and micropowders may be governed by the specifics of the dynamic compression of nanoparticles. However, it is not possible to make further considerations of the n value without a proper study of the steady conditions for nNi. This may be an interesting task for future research.

## 6. Conclusions

The response of samples of pressed nickel nanoparticles with ~50% porosity to the uniaxial shock wave loading at the range of 1.6–7.1 GPa is complex. At these pressures, a shock wave entering the material splits into a precursor and compaction wave. In the experiments, this structure may be noticeably affected by precursor reverberations propagating between the investigated surface of the sample and the compaction wave front. The two-wave structure, reverberations, and their effect on the measurements were described using the joint analysis of a wave profile, distance–time, and pressure–particle velocity diagrams. Evidence of precursor attenuation was also observed.

The obtained Hugoniot and rise time data demonstrate that the pore size is probably not the key parameter that defines the shock wave properties of the studied nanoporous material. The most notable result is that in the studied pressure range, the rise times of the compaction waves propagating in the nanosized nickel powder were comparable to those measured for the microsized powders. This agreement leads to the fact that the thicknesses of the compaction waves in the case of the nanoporous nickel are proportional to several hundred average particle sizes rather than an average particle size, which is typical for various micropowders.

## Figures and Tables

**Table 1:** Parameters of experiments.

No.	Impactor Material	Window Material	Reflective Foil Thickness, μm	Impactor Velocity V_i_, m/s	Sample Thickness *h_s_*, mm
1	Copper	water	7	296	2.10
2	D16t aluminum alloy	LiF	7	587	2.01
3		water	7	648	2.125
4		water	7	682	2.98
5		water	7	837	2.06
6		water	50	1010	5.44
7.1		water	50	1010	3.675
7.2		LiF	50		2.96
8.1		no window	10	1105	3.91
8.2		water	10		3.87
8.3		LiF	10		3.82
9.1		water	100	1260	3.44
9.2		LiF	20		3.33

**Table 2:** Hugoniot and wave front data for precursor.

No.	Wave Velocity *U_s_*_1_, km/s	Particle Velocity *u_p_*_1_, km/s	Pressure *p*_1_, GPa	Strain Rate ${\dot{\varepsilon }}_{1},\;\mu s^{-1}$	Rise Time *τ*_1_, μs
1	2.442	0.040	0.46	0.15	0.10
2	1.951	0.073	0.42	1.75	0.04
3	2.457	0.043	0.49	2.67	0.08
4	2.492	0.021	0.24	0.10	0.17
5	2.604	0.040	0.49	0.31	0.09
6	2.599	0.023	0.28	-	-
7.1	2.631	0.020	0.25	-	-
7.2	1.726	0.038	0.31	-	-
8.1	1.753	0.038	0.30	0.54	0.14
8.2	2.037	0.017	0.15	0.09	0.16
8.3	-	-	-	-	-
9.1	2.581	0.022	0.25	-	-
9.2	-	-	-	-	-

**Table 3:** Hugoniot and wave front data for compaction wave.

No.	Wave Velocity *U_s_*_2_, km/s	Particle Velocity *u_p_*_2_, km/s	Pressure *p*_2_, GPa	Strain Rate ${\dot{\varepsilon }}_{2},\;\mu s^{-1}$	Rise Time *τ*_2_, μs
1	2.442	0.250	1.63	0.33	-
2	-	-	-	-	-
3	1.219	0.468	2.815	2.67	-
4	1.202	0.500	2.85	51.31	0.034
5	1.442	0.579	4.11	101.60	0.014
6	1.905	0.651	5.84	-	-
7.1	1.504	0.700	4.99	-	-
7.2	0.903	0.790	3.47	-	-
8.1	1.498	0.781	5.34	64.59	0.006
8.2	1.592	0.768	5.57	- *	- *
8.3	1.450	0.784	5.28	78.45	0.010
9.1	1.779	0.846	6.85	-	-
9.2	1.900	0.830	7.11	-	-

* These values were not calculated due to issues with the VISAR data analysis.

## Data Availability

The data that support the findings of this study are available from the corresponding author upon reasonable request.
